# Horizons in evolutionary genomics: an interview with David Ferrier

**DOI:** 10.1186/s12915-018-0587-3

**Published:** 2018-11-01

**Authors:** David E. K. Ferrier

**Affiliations:** The Scottish Oceans Institute, Gatty Marine Laboratory, School of Biology, University of St. Andrews, St. Andrews, UK

**Keywords:** Evolution, Development, Genomics, Homeobox, Genome organization, Phylogenetics

## Abstract

David Ferrier is a Reader at the University of St Andrews and Deputy Director of the Scottish Oceans Institute, where his lab studies how the diversity of form in the animal kingdom evolved, with an emphasis on using comparative genomics. In this interview, David shares his thoughts on how to escape the ‘straitjacket’ of traditional model systems, transparency in peer review, and the past and future of genome sequencing.

## What are your current research interests?

I take a comparative genomics approach to study animal evolutionary developmental biology, which could be summarized as evolutionary developmental genomics. I tend to focus on the homeobox-containing genes since there are so many intriguing instances of links between their organization in animal genomes and their functions (most famously in the case of the Hox gene cluster). They tend to provide a good indication of changes that happen at major transitions in the animal kingdom, such as whole genome duplications or major rearrangements and departures from the deeply conserved synteny (gene neighborhoods) that have been one of the most startling findings in this era of whole genome sequencing [1–7].
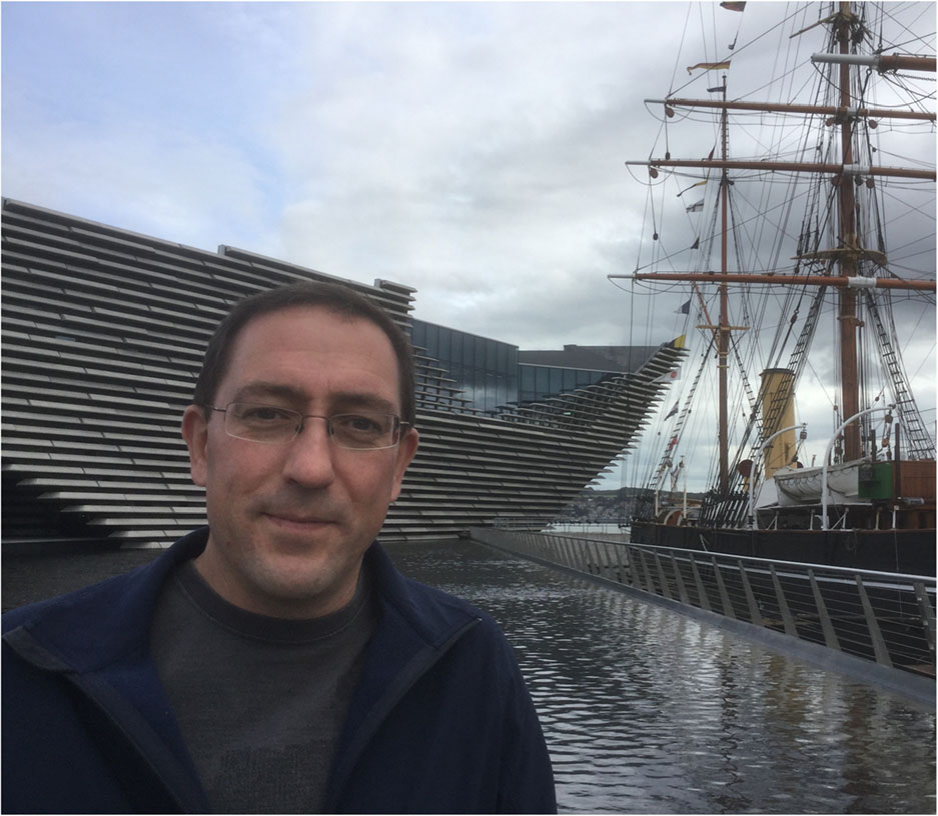


## What are your predictions for the field over the next 5 years?

The technical developments in DNA sequencing are progressing at a staggering rate, and costs are tumbling. This is a very exciting time to be working in fields associated with genomics. I think some of the key areas of progress that are happening (but that we need more of) are things like improved taxon sampling, to provide denser coverage of clades as well as wider coverage of the animal kingdom (speaking as a zoologist!); improvements to assembly pipelines, which are also being aided by things like long-molecule sequencing technologies; and developments in annotation tools and pipelines, as plenty of manual annotation is still required for those of us interested in the precise details of gene family evolution and organization.

CRISPR and RNAi techniques have also been truly revolutionary, and this now opens up biology to make use of broader taxon sampling so that we can start to move away from the straitjacket of a small handful of model organisms. Instead we can find the organism that has the interesting biology and do the experiments in that species, rather than having to adapt our research so that we have to do experiments in one of the big traditional systems like *Drosophila*, *Caenorhabditis*, or one of the few vertebrates that are usually worked on for functional genetics. The research funding bodies have a major role to play in this new era of using a diversity of species in biology involving functional genetics, and it is to be hoped that they do respond by broadening their horizons.

## What motivates you to provide peer review for journals?

It is an essential element of science, and so a duty of all scientists to contribute. There are also more selfish sides to agreeing to review manuscripts, such as seeing interesting work before the community at large as well as helping to shape this work, hopefully for the benefit of the field.

## What changes, if any, would you make to the current system of peer review?

There are interesting developments in more open peer review, exploring different forms of this (e.g. review reports published either with or without the reviewers’ names). There are pros and cons [8] and having experienced various versions of open review I’m not sure if there is one best option, but on balance this extra transparency is likely to lead to overall improvements.

I am wary about making this second point, because as an English speaker I am very lucky that the international language of science is English. And I recognize that things are much harder for those who have English as a second language. So this comment is certainly not meant as a criticism of those who might struggle to write well in English and I certainly do not want to give the impression that I am criticizing the writing of non-English speakers. Nothing could be further from the truth. My point though is that there is often a scope for manuscripts to be improved for written English before going out to academic editors and referees.

The aim must be to enable academic editors and reviewers to focus solely on the science, making the process more efficient. Time is precious, and it can be frustrating within the peer review process when one feels the need to provide lots of comments on sentence construction and appropriate vocabulary. Also, part of the problem is that the professional editing services that authors have to pay large sums of money for are of highly variable quality. Whether the burden for this extra language editing should be picked up more by the journals is a moot point, but there is certainly scope for more to be done by the universities and research institutions around the world, to develop further their in-house editing services.

## Have you had any memorably good or bad experiences of peer review, as an author or as a reviewer?

I think we all have occasional bad experiences, which usually center on feelings of injustice involving editors or reviewers not seeming to read the manuscript carefully enough. Although a counter-argument would be to improve the clarity of writing! I once had a manuscript turned away from *BMC Biology* because a molecular phylogeny in our paper did not have enough taxa to resolve the position of the species we were working on with any real confidence. But in fact that paper was nothing to do with the phylogenetic position, and instead was merely illustrating branch lengths as a minor component of the main story. Conversely, I did once have the dream situation of receiving referees’ comments that required no revisions at all! So it is all a ‘mixed bag’ really, but when there are problems, appeals processes and talking to editors can help, although this can seem daunting and editors handle this in very different ways in terms of their willingness to engage with such communications.

On the reviewing side, I once had the bizarre experience of dealing with a manuscript that turned out to be from some creationists. In my report I focused on the shockingly poor writing and incoherent organization of the rambling manuscript, with no clear message and extensive inappropriate plagiarism. Not once did I comment on the evolutionary angle, because so much else was wrong with the manuscript, but after the authors received the comments they ranted about narrow-minded, establishment scientists and completely missed the point of my comments. It was at this point that I looked into the backgrounds of the authors more carefully and discovered that they gave their affiliation as a creationist/intelligent design institute (I try to referee without taking into account the author’s name, reputation, and affiliation too much, at least in the first instance). Following the authors’ ranting response the journal editor then stepped in and terminated the whole process and rejected the manuscript, I’m relieved to say.

**Website:** https://risweb.st-andrews.ac.uk/portal/en/persons/david-ellard-keithferrier(9d113045-bca1-49ef-8315-05b2d8425d14).html and https://synergy.st-andrews.ac.uk/edge/.
